# Supplementary material for: Room-temperature mechanocaloric effects in lithium-based superionic materials
Source: Nat Commun. 2018 Aug 20;9:3337. doi: 10.1038/s41467-018-05835-9 (PMC6102246; doi:10.1038/s41467-018-05835-9)
Supplement: Supplementary file 1 — Supplementary Information [file 41467_2018_5835_MOESM1_ESM.pdf]

SUPPLEMENTARY FIGURES

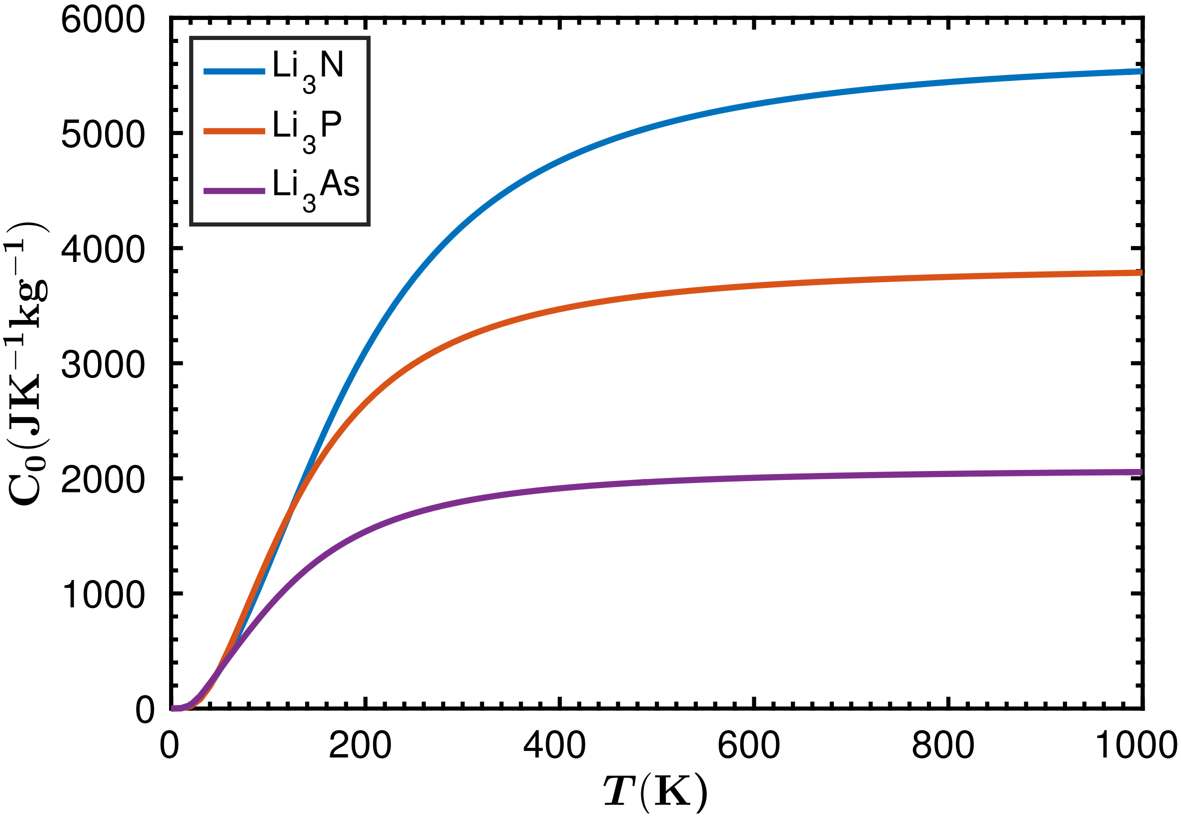

Supplementary Figure 1: Heat capacity of Li_3N, Li_3P, and Li_3As calculated at the corresponding equilibrium volumes and expressed as a function of temperature. Calculations were performed with first-principles methods based on density functional theory and the quasi-harmonic approximation [10–12].

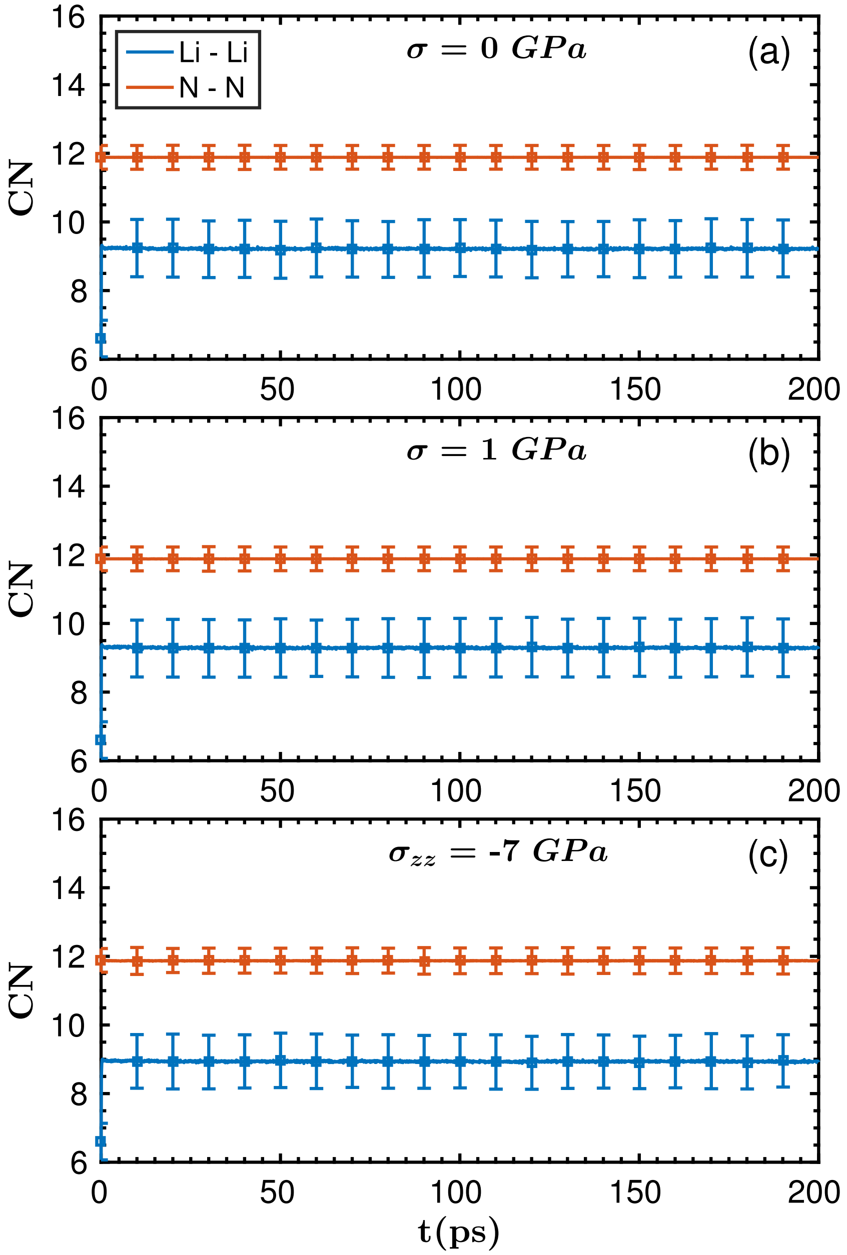

Supplementary Figure 2: Li–Li and N–N coordination numbers in Li_3N calculated during molecular dynamics simulations at $T = 300$ K and considering different mechanical stress conditions. The value of the coordination numbers remain constant along the whole simulation which indicates the absence of any σ-induced structural phase transition.

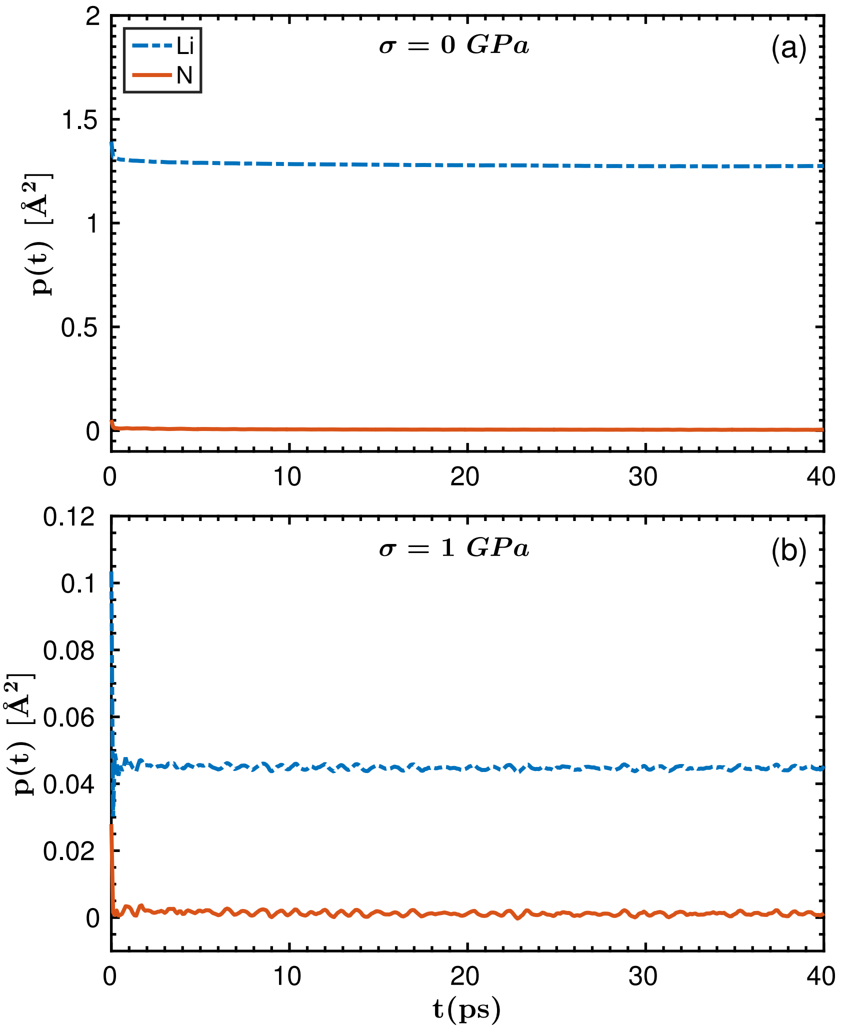

Supplementary Figure 3: Atomic position correlation function in Li_3N calculated at $T = 300$ K and different hydrostatic pressure conditions. The position correlation function is defined as $p_i(t) = \langle (\mathbf{r}(t+t_0) - \mathbf{R}^0) \cdot (\mathbf{r}(t_0) - \mathbf{R}^0) \rangle$, where $\mathbf{R}^0$ correspond to the perfect-lattice positions of atoms belonging to species i and $\langle \cdots \rangle$ denotes average over t_0's and ions [5,6]. The sublattice formed by i atoms is vibrationally stable if $p_i(t \to \infty) = 0$ since the displacements at widely separated times become uncorrelated; otherwise, if the atoms acquire a permanent vibrational displacement, $p_i(t \to \infty) \neq 0$. Our results show that the sublattice formed by N atoms remains vibrationally stable thus indicating the absence of any σ-induced structural phase transition (mind that as the system is superionic $p_{\mathrm{Li}}(t) \neq 0$).

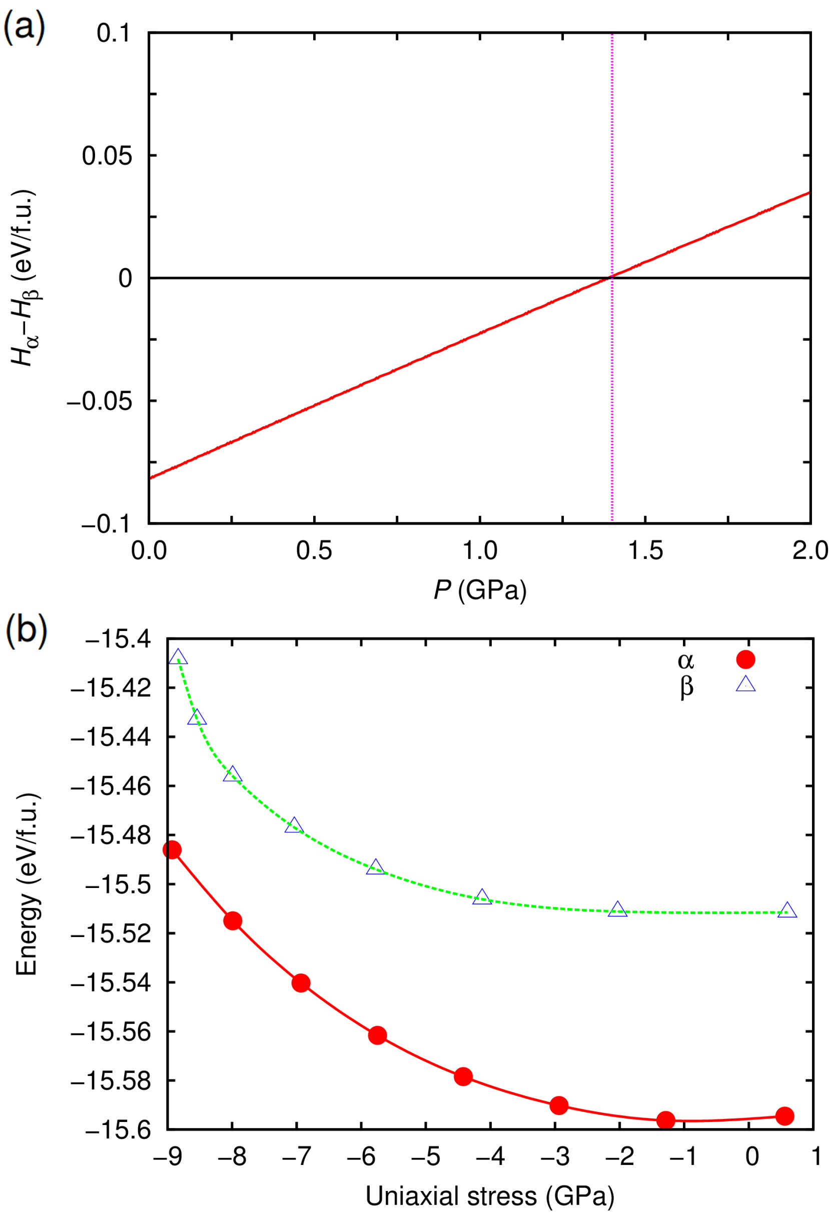

Supplementary Figure 4: Enthalpy and energy differences between the α and β Li_3N polymorphs calculated at different stress conditions. Hydrostatic pressure (a) and tensile uniaxial stress (b). Enthalpy calculations (a) indicate a pressure–induced phase transformation between the two polymorphs occurring at $P_t \approx 1.4$ GPa; this result is consistent with previous theoretical and experimental reports [13–15] and shows that P_t is safely out of the hydrostatic–pressure range considered in the present study (namely, $P \leq 1$ GPa). Energy calculations (b) indicate the absence of any phase transformation between the α and β polymorphs as induced by tensile uniaxial stresses below $|\sigma_{zz}| \sim 10$ GPa.

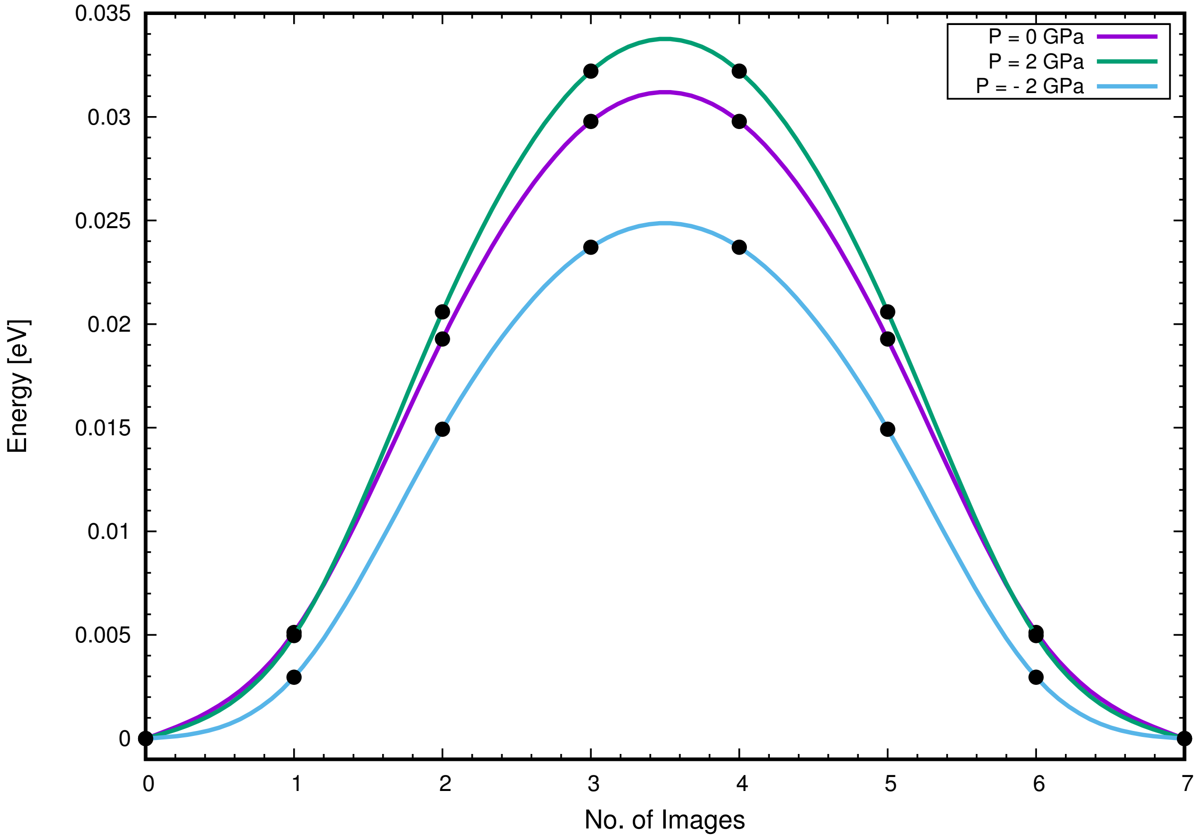

Supplementary Figure 5: Kinetic energy barrier for Li diffusion between two equivalent interstitial positions in Li_3N calculated as a function of hydrostatic pressure. Positive (negative) pressures induce a reduction (expansion) in the volume of the system that in turn provokes an increase (reduction) in the Li-diffusion energy barrier (i.e., maximum value in the energy curves) as compared to equilibrium.

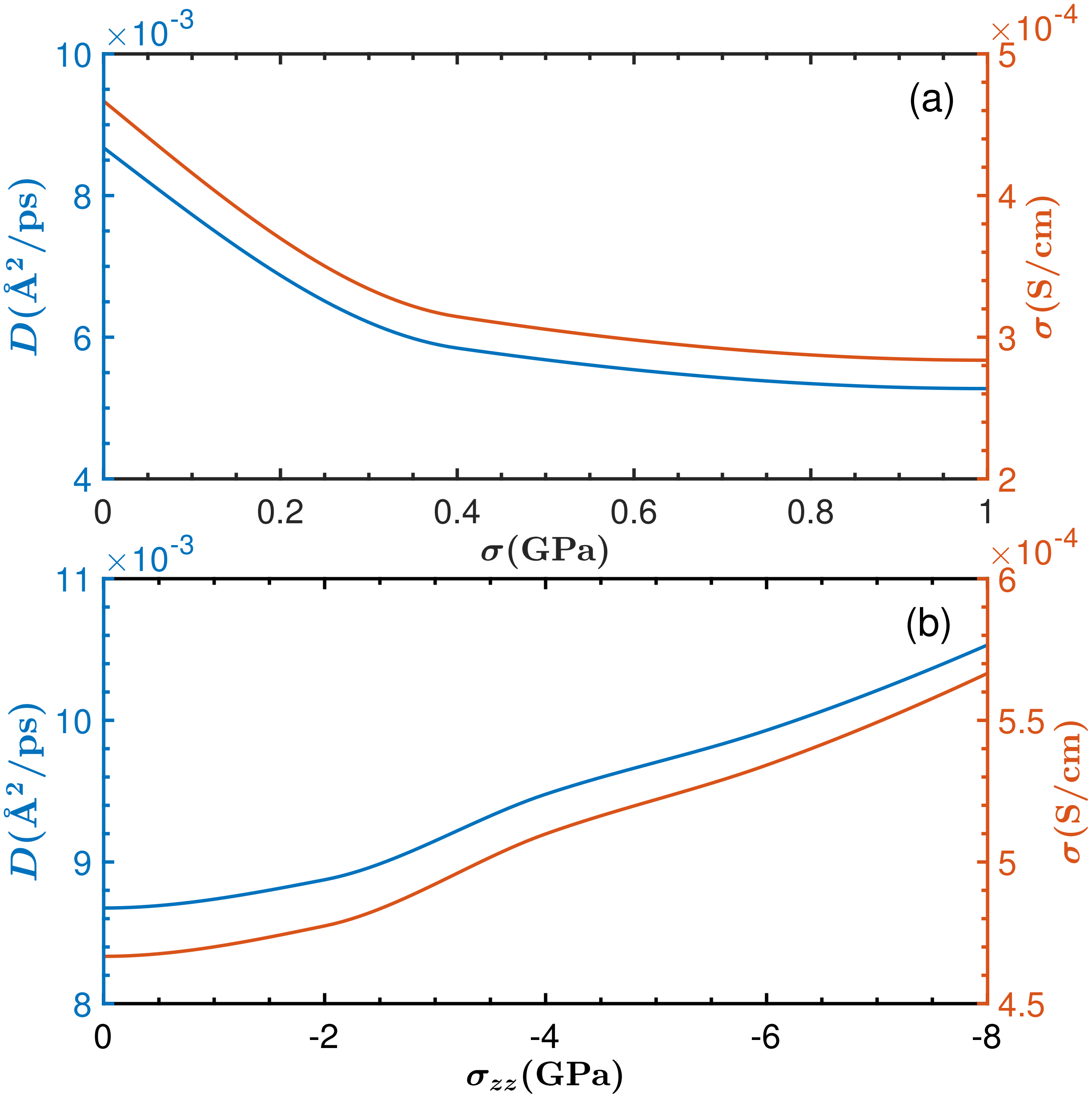

Supplementary Figure 6: Lithium diffusion coefficient and corresponding ionic conductivity in Li_3N at $T = 300$ K. Properties are calculated as a function of hydrostatic pressure (a) and tensile uniaxial stress (b). An increase in hydrostatic pressure (tensile uniaxial stress) provokes depletion (enhancement) of the ionic transport properties.

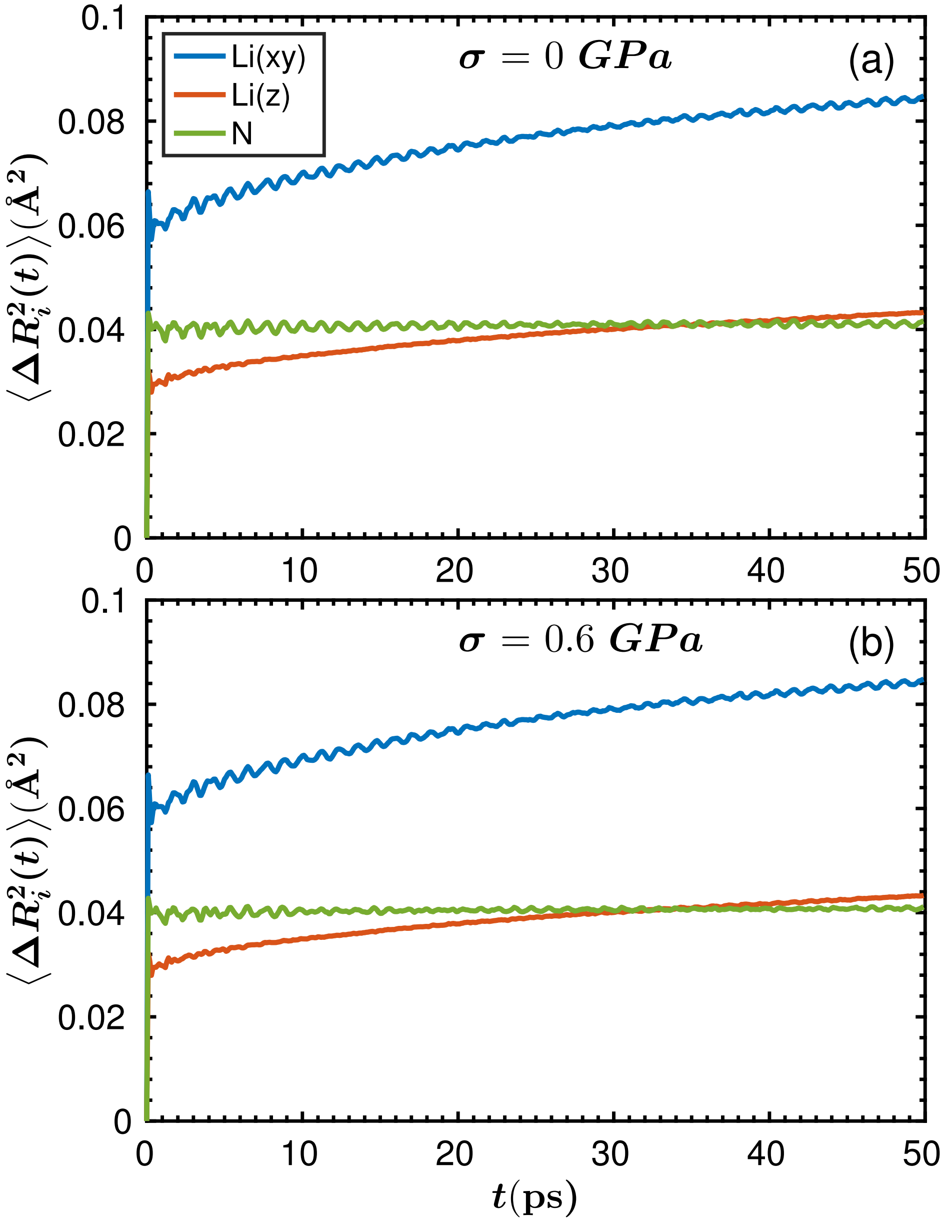

Supplementary Figure 7: Room-temperature mean squared displacement of bulk Li_3N. Calculated at equilibrium (a) and under pressure (b) with classical molecular dynamics and expressed as a function of simulated time and Li^+ diffusion direction, namely, within the Cartesian x–y plane and along the Cartesian z direction (which is equivalent to the hexagonal c–axis). The slopes of the curves estimated within the x–y plane and along the z direction are quite similar in both cases. The characteristic time scale of superionic switching is shown to be of just few picoseconds, which is consistent with ultrafast x-ray spectroscopy observations [16].

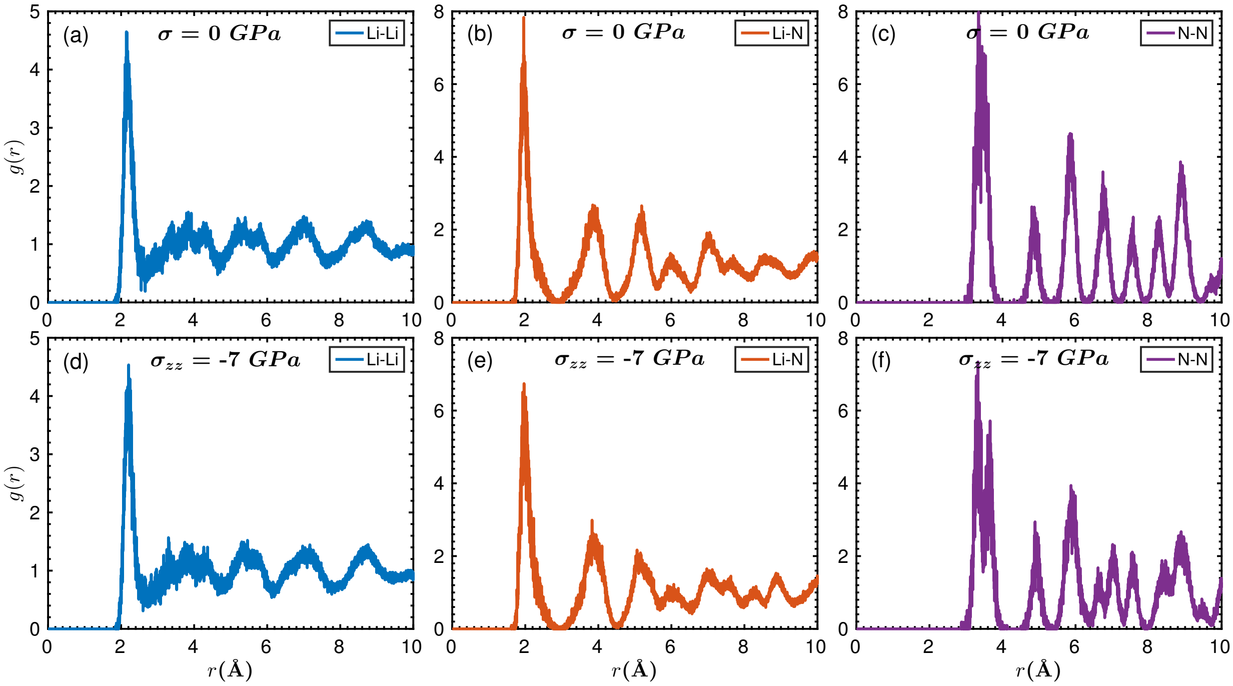

Supplementary Figure 8: Radial pair distribution function in Li_3N. Calculated at equilibrium and under a tensile load of -7 GPa for the (a)-(d) Li–Li, (b)-(e) Li–N, and (c)-(f) N–N ionic pairs at $T = 300$ K. Corresponding $g(r)$ profiles present almost identical structural traits independently of the applied uniaxial stress, which demonstrates the absence of any σ-driven structural phase transition.

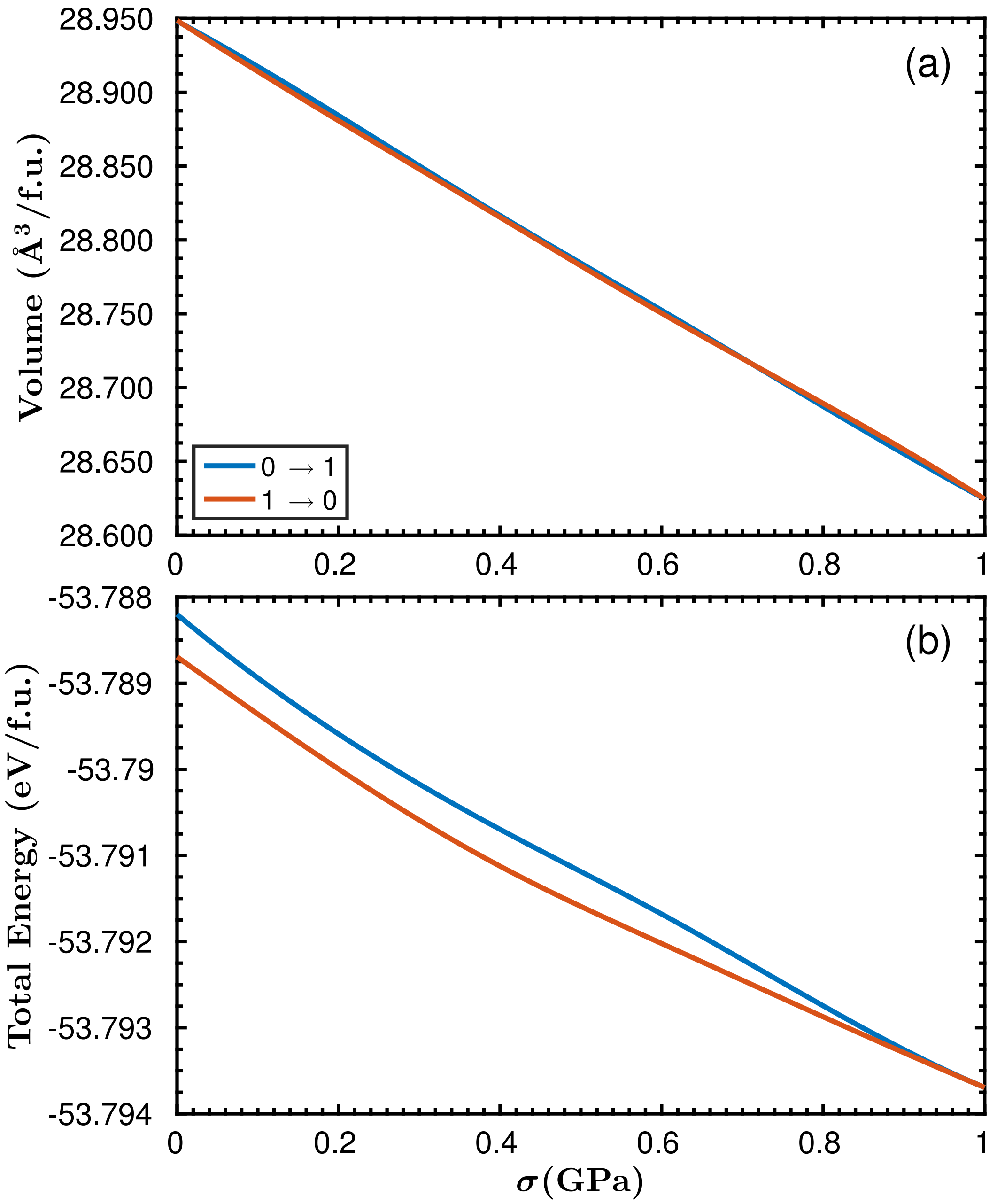

Supplementary Figure 9: Assessment of reversibility features associated to fast-ion conductors. Volume (a) and total energy (b) of Li_3N calculated during a long molecular dynamics run of a total duration of $2,200$ ps in which the applied hydrostatic pressure is smoothly and constantly varied from 0 to 1 GPa and then backwards from 1 to 0 GPa at a fixed temperature of 300 K. The minuteness of the differences between the volumes and energies corresponding to a same thermodynamic state in the "$0 \to 1$" and "$1 \to 0$" series, demonstrates the highly reversible nature of the physical changes inflicted by σ on superionic Li_3N.

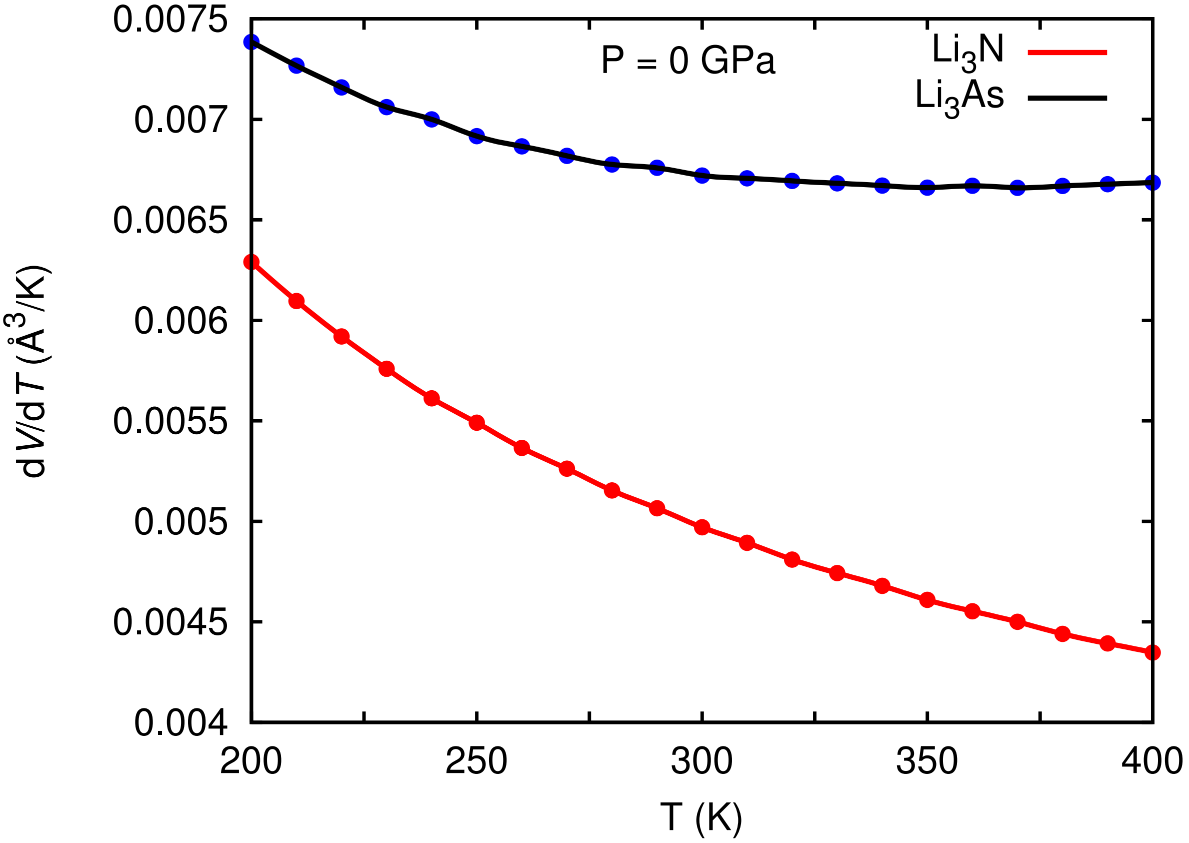

Supplementary Figure 10: Temperature derivative of the volume calculated in Li_3N and Li_3As at $T = 300$ K by means of the quasi-harmonic approximation [10–12] and expressed as a function of temperature. Li_3As is shown to be elastically softer than Li_3N. This finding along with the heat capacity results shown in Supplementary Figure 1 suggest that it is possible to obtain larger adiabatic temperature shifts than reported for pure Li_3N by means of N–site doping strategies involving isoelectronic and larger-radius/larger-mass ions.

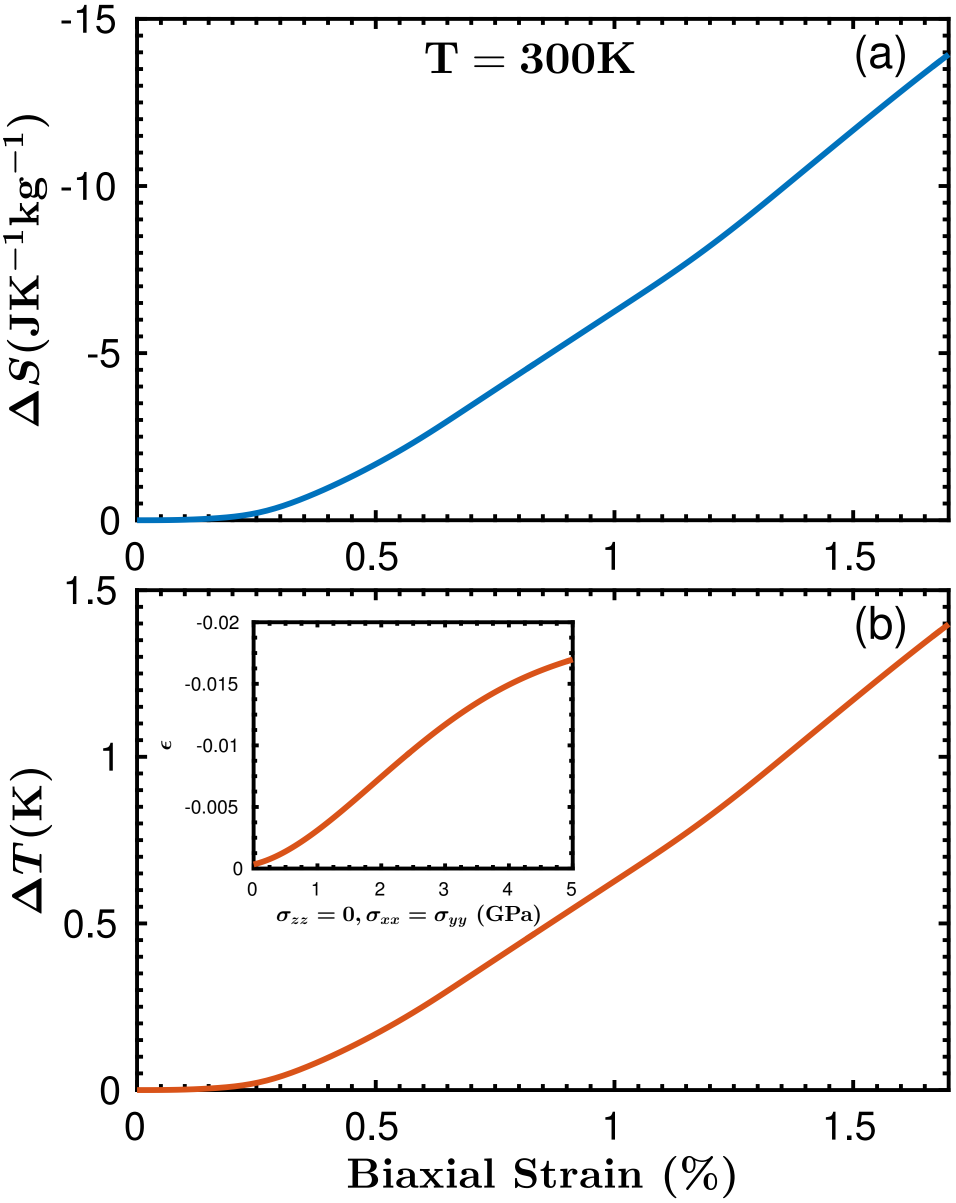

Supplementary Figure 11: Mechanocaloric effects induced by compressive biaxial stresses. (a) Isothermal entropy and (b) adiabatic temperature shift results obtained by considering several compressive biaxial stresses at $T = 300$ K. The relationship between biaxial compressive stress and strain deformation along the Cartesian x and y directions is shown in the inset. Compressive biaxial stresses induce direct mechanocaloric effects in Li_3N that are analogous to those found in the hydrostatic case.

SUPPLEMENTARY TABLES

Supplementary Table I: BMH interatomic pairwise potential parameters used to describe Li_3N in molecular dynamics simulations [1]. $Z_{\mathrm{Li}} = +1$ and $Z_{\mathrm{N}} = -3$ in e units.

	A (eV)	ρ (Å)	C (eV Å^6)
Li – N	232.59366	0.43284	0
Li – Li	916.5397	0.14270	0
N – N	22259.27515	0.28200	2102.66667

Supplementary Table II: Giant mechanocaloric (MC) effects near room temperature. T represents working temperature, $|\sigma|$ applied mechanical stress, $|\Delta S|$ isothermal entropy change, $|\Delta T|$ adiabatic temperature change, $|\Delta T|/|\sigma|$ mechanocaloric strength, SMA shape-memory alloy, FE ferroelectric, OIH organic-inorganic hybrid perovskite, and FIC fast-ion conductor. $|\Delta T|$ values have been obtained by using zero-pressure specific heat capacities. Corresponding references can be found in works [3,7,17,18]. The reported giant MC effects are originated by structural phase transformations, made the exception of Li_3N.

Giant MC material	T (K)	$\|\sigma\|$ (GPa)	$\|\Delta S\|$ ($JK^{-1}Kg^{-1}$)	$\|\Delta T\|$ (K)	$\|\Delta T\|/\|\sigma\|$ (K GPa^{-1})	Material type
NiTi	295	0.65	–	25.5	39.3	SMA
$Ni_{49.26}Mn_{36.08}In_{14.66}$	293	0.26	24.0	4.5	17.3	SMA
$Fe_{49}Rh_{51}$	308	0.11	12.5	8.1	73.6	SMA
$Cu_{68}Zn_{16}Al_{16}$	300	0.28	16.0	15.0	53.6	SMA
$BaTiO_3$	300	6.50	8.0	5.5	0.9	FE
$(NH_4)_2SO_4$	219	0.10	60.0	8.0	80.0	FE
$[TPrA][Mn(dca)_3]$	330	0.01	30.5	4.1	505.1	OIH
AgI	400	0.25	62.0	36.0	144.0	FIC
Li_3N	300	1.00	32.0	2.8	2.8	FIC

SUPPLEMENTARY METHODS

The interatomic potential adopted to investigate Li_3N with molecular dynamics (MD) simulations is of the Born-Mayer-Huggins (BMH) form:

$$V_{ij}(r) = A_{ij} e^{-\frac{r}{\rho_{ij}}} - \frac{C_{ij}}{r^6} + \frac{Z_i Z_j}{r} \ , \tag{1}$$

where subscripts i and j represent the ionic species in the system, r the radial distance between a couple of atoms, and the corresponding parameter values are reported in Supplementary Table 1 [1]. Each pairwise interatomic term is composed of three different contributions; the first one is of exponential type and accounts for the short-ranged atomic repulsion deriving from the overlapping between electron clouds; the second term is proportional to r^{-6} and represents the long-ranged atomic attraction due to dispersive van der Waals forces; the third term is the usual Coulomb interaction between puntual atomic charges.

In order to accurately compute the thermodynamic shifts $\Delta S(\sigma, T)$ and $\Delta T(\sigma, T)$ induced by compressive hydrostatic and tensile uniaxial stresses in bulk Li_3N, we calculated the corresponding volumes, uniaxial strains, and heat capacities (C_σ) over dense grids of (σ, T) points spaced by $\delta\sigma = 0.1$ GPa and $\delta T = 20$ K [2–4]. Mechanical strains were estimated directly from the molecular dynamics simulations, and heat capacities from the differences between averaged internal energies obtained in simulations performed at T and $T \pm 20$ K conditions. Spline interpolations were subsequently applied to the calculated sets of volume and uniaxial strain data points in order to obtain continuous and well-behaved functions for an accurate determination of the corresponding temperature and stress derivatives. The ΔS and ΔT values appearing in Figs. 2-5 of the main text were calculated by numerically integrating those functions with respect to the applied hydrostatic and uniaxial stresses, and by considering $C_\sigma(\sigma, T) \approx C_\sigma(0, T) \equiv C_0(T)$.

Ab initio molecular dynamics (AIMD) simulations based on density functional theory were performed in the canonical (N, V, T) ensemble for bulk Li_3N by considering both the α and β polymorphs. The objective of these calculations was to assess the reliability of the employed BMH interatomic potential model in our molecular dynamics simulations. The temperature in the AIMD simulations was kept fluctuating around a set-point value by using Nose-Hoover thermostats. Large simulation boxes containing up to 256 atoms were used in all the cases, and periodic boundary conditions were applied along the three corresponding

Cartesian directions. Newton's equations of motion were integrated using the customary Verlet's algorithm and a time-step length of 10^{-3} ps. Γ-point sampling for integration within the first Brillouin zone was employed in all the AIMD simulations. The calculations comprised long simulation times of up to $\sim$ 90 ps. We focused on the description of the superionic features which were identified via inspection of the mean squared displacement function estimated directly from the AIMD runs. All our test calculations, both AIMD and classical MD simulations, were carried out considering $T \neq 0$ and zero-pressure conditions. We consistently found that stoichiometric β–Li_3N becomes superionic at $T_s^{\rm DFT} = 1000(100)$ K in the AIMD simulations and at $T_s^{\rm BMH} = 900(50)$ K in the MD simulations. Likewise, the superionic transition temperature obtained in stoichiometric α–Li_3N with DFT methods is $T_s^{\rm DFT} = 400(100)$ K, and with classical molecular dynamics $T_s^{\rm BMH} = 300(50)$ K (figures within parentheses indicate the corresponding numerical uncertainties). Therefore, we may conclude that the adopted BMH interaction potential model appears to be physically reliable in the description of the ionic-transport properties of Li_3N as it provides results that are consistent with first-principles methods.

It is worth noting that the type of computational approach adopted in this study certainly is able to reproduce very accurately the ion-transport, energy, and structural properties of fast-ion conductors under mechanical stresses (see, for instance, Refs. [2,4–6]). Moreover, computational approaches are increasingly being applied with success to the analysis and prediction of mechanocaloric effects in materials (see, for instance, Refs. [3,7–9]) since these are very reliable and can be employed in a very effective and inexpensive manner.

SUPPLEMENTARY REFERENCES

[1] Wolf, M. L., Walker, J. R. & Catlow, C. R. A. A molecular dynamics simulation study of the superionic conductor lithium nitride: I. *J. Phys. C: Sol. Stat. Phys.* **17**, 6623 (1984).

[2] Cazorla, C & Errandonea, D. Giant mechanocaloric effects in fluorite-structured superionic materials. *Nano Lett.* **16**, 3124 (2016).

[3] Sagotra, A. K., Errandonea, D. & Cazorla, C. Mechanocaloric effects in superionic thin films from atomistic simulations. *Nat. Commun.* **8**, 963 (2017).

[4] Sagotra, A. K. & Cazorla, C. Stress-mediated enhancement of ionic conductivity in fast-ion conductors. *ACS Appl. Mater. Interfaces* **9**, 38773 (2017).

[5] Cazorla, C. & Errandonea, D. Superionicity and polymorphism in calcium fluoride at high pressure. *Phys. Rev. Lett.* **113**, 235902 (2014).

[6] Cazorla, C., Sagotra, A. K., King, M. & Errandonea, D. High-pressure phase diagram and superionicity of alkaline-earth metal difluorides. *J. Phys. Chem. C* **122**, 1267 (2018).

[7] Aznar, A., Lloveras, P., Romanini, M., Barrio, M., Tamarit, J. Ll., Cazorla, C., Errandonea, D., Mathur, N. D., Planes, A., Moya, X. & Mañosa, Ll. Giant barocaloric effects over a wide temperature range in superionic conductor AgI. *Nat. Commun.* **8**, 1851 (2017).

[8] Stern-Taulats, E., Castán, T., Planes, A., Lewis, L. H., Barua, R., Pramanick, S., Majumdar, S. & Mañosa, Ll. Giant multicaloric response of bulk $Fe_{49}Rh_{51}$. *Phys. Rev. B* **95**, 104424 (2017).

[9] Zarkevich, N. A., Johnson, D. D. & Pecharsky, V. K. High-throughput search for caloric materials: the CaloriCool approach. *J. Phys. D: Appl. Phys.* **51**, 024002 (2017).

[10] Kresse, G., Furthmüller, J. & Hafner, J. Ab initio force constant approach to phonon dispersion relations of diamond and graphite. *Europhys. Lett.* **32**, 729 (1995).

[11] Alfè, D. PHON: A program to calculate phonons using the small displacement method. *Comp. Phys. Commun.* **180**, 2622 (2009).

[12] Cazorla, C. & Íñiguez, J. Insights into the phase diagram of bismuth ferrite from quasiharmonic free-energy calculations. *Phys. Rev. B 88*, 214430 (2013).

[13] Cui, S., Feng, W., Hu, H., Feng, Z. & Wang, Y. Structural transition of Li_3N under high pressure: A first-principles study. *Solid State Commun.* **149**, 612 (2009).

[14] Yan, Y., Zhang, J. Y., Cui, T., Li, Y., Ma, Y. M., Gong, J., Zong, Z. G. & Zou, G. T. First-principles study of high pressure phase transformations in Li_3N. *Eur. Phys. J. B* **61**, 397

(2008).

[15] Ho, A. C., Granger, M. K. & Ruoff, A. L. Experimental and theoretical study of Li_3N at high pressure. *Phys. Rev. B* **59**, 6083 (1999).

[16] Miller, T. A., Wittenberg, J. S., Wen, H., Connor, S., Cui, Y. & Lindenberg, A. M. The mechanism of ultrafast structural switching in superionic cooper (I) sulphide nanocrystals. *Nat. Commun.* **4**, 1369 (2013).

[17] Bermúdez-García, J. M., Sánchez-Andújar, M., Castro-García, S., López-Beceiro, J., Artiaga, R. & Señarís-Rodríguez, M. A. Giant barocaloric effect in the ferroic organic-inorganic hybrid [TPrA][Mn(dca)$_3$] perovskite under easily accessible pressures. *Nat. Commun.* **8**, 15715 (2017).

[18] Bermúdez-García, J. M., Sánchez-Andújar, M. & Señarís-Rodríguez, M. A. A new playground for organic-inorganic hybrids: barocaloric materials for pressure-induced solid-state cooling. *J. Phys. Chem. Lett.* **8**, 4419 (2017).
